# Healthy eating index (HEI) and stroke: Population-level clinical evidence

**DOI:** 10.1016/j.neuros.2025.100019

**Published:** 2025-12-30

**Authors:** Jinpyo Hong, Manuel Bita, Alain Lekoubou, Djibril M Ba

**Affiliations:** a Penn State College of Medicine, Penn State Health Milton S. Hershey Medical Center, Hershey, PA 17033; b Georgia State University, Atlanta, GA 30302; c Department of Neurology, Penn State College of Medicine, Penn State Health Milton S. Hershey Medical Center, Hershey, PA 17033; d Department of Public Health Sciences, Penn State College of Medicine, Penn State Health Milton S. Hershey Medical Center, Hershey, PA 17033

Dear Editor,

Unhealthy eating habits is associated with increased risk of death in the U.S [1]. Stroke is frequent and, despite significant therapeutic progress, remains the fifth cause of death in the U.S [2]. In addition, eating habits are often discussed as one of the pillars of maintaining good health and preventing chronic non-communicable diseases [3]. However, there is a dearth of data on the association between unhealthy eating habits and mortality in stroke patients. A recent prospective cohort study conducted by Tirani et al. discusses the relationship between healthy lifestyle score and incident cardiovascular disease, but the study does not specifically focus on eating habits, nor are the patient cohorts comprised of patients with stroke as a baseline diagnosis [4]. Understanding the limited availability of literature, this study aims to investigate the association between Healthy Eating Index (HEI)-2015 and all-cause and cause-specific mortality risk in the U.S.

We conducted a prospective cohort study using the publicly released de-identified of the National Health and Nutrition Examination Survey (2003–2018). All-cause and cause-specific mortality were assessed in all participants linked to the National Death Index (NDI) mortality data using death certificates through December 31, 2019. Our study population consisted of adults (18+ years old) of self-reported physician diagnosis of stroke during the NHANES surveys. Dietary data was collected using 24-hour dietary recalls. For each participant, the person-time was calculated as the time from the baseline survey participation interview date until the date of death or end of follow-up (December 31, 2019), whichever came first. The exposure was healthy eating habits, which was evaluated using the HEI-2015. We stratified the HEI-2015 scores into 3 categories (≥80: “good” diet; 51–79: “fair” diet; ≤50: “poor” diet) [5]. The outcomes of interest were all-cause mortality, cardiovascular mortality, and stroke mortality, identified using the International Classification of Diseases, 10th Revision codes (ICD-10). We used Cox proportional hazards regression models to calculate multivariable-adjusted hazard ratios (HRs) and 95 % confidence intervals (95 % CIs) for all-cause and cause-specific mortality, and the following potential confounders were selected and adjusted for in the multivariable Cox proportional regression models: age, race/ethnicity, sex, marital status, education, socio-economic variable such as poverty-income ratio (PIR), and total energy intakes [6].

A total of 1498 individuals had a self-reported diagnosis of stroke. The mean age of stroke patients with a poor, fair, and good diet was 60, 68, and 74 years old, respectively. A total of 620 deaths were recorded (41.3 % mortality of self-reported stroke patients). Within 620 all-cause deaths, 181 deaths (29.2 % of all-cause deaths and 12.0 % mortality of self-reported stroke patients) were related to cardiovascular diseases, while 53 deaths were stroke deaths (8.6 % of all-cause deaths and 3.5 % mortality of self-reported stroke patients). Table 1 shows the association between HEI-2015 and mortality. A good or fair HEI was not associated with a lower all-cause, cardiovascular, or stroke mortality.

In this nationally representative sample of stroke patients, we found no association between healthy eating habits and all-cause, cardiovascular, and stroke mortalities. Previous studies using the HEI-2015 in the general population have reported an inverse association between individual dietary scores and all-cause mortality [7]. Although we found similar inverse trends, Our study did not yield any significant provides data on the association between HEI-2015 and were all-cause mortality, cardiovascular mortality, and stroke mortality in this a particular population of stroke patients. Simultaneously, there were studies that showed contrary results. Liu et al.’s study exhibited 26 % lower all-cause mortality in patient cohort with highest quartile of Gut Microbiota Dietary Index (DI-GM) [8]. While this study makes a significant contribution to the limited existing literature, we acknowledge that our study may have been underpowered to detect such a significant association and might not accurately depict the interrelated values of social determinants of health not covered by the adjustments made during statistical analyses, which could potentially play a role in the results we obtained.

## Figures and Tables

**Table 1:** Association between HEI-2015 and Mortality.

Mortality type	Fair diet vs. poor diet: Hazard ratios (95 % confidence interval)	Good diet vs. poor diet: Hazard ratios (95 % confidence interval)
**All-cause**	0.92 (0.73–1.17)	0.58 (0.27–1.24)
**Cardiovascular**	0.94 (0.61–1.45)	0.56 (0.10–3.07)
**Stroke**	1.20 (0.61–2.39)	0.43 (0.04–4.30)
